# Dietary phospholipid carriers of DHA do not increase brain DHA levels: a replication study

**DOI:** 10.1016/j.jlr.2025.100913

**Published:** 2025-09-26

**Authors:** Brinley J. Klievik, Yan Fu, Aidan D. Tyrrell, Chuck T. Chen, Adam H. Metherel, Richard P. Bazinet

**Affiliations:** Department of Nutritional Sciences, Temerty Faculty of Medicine, University of Toronto, Toronto, Ontario, Canada

**Keywords:** fatty acid metabolism, omega-3 fatty acids, brain lipids, lysophosphatidylcholine, phosphatidylcholine

## Abstract

DHA is primarily found in fish and seafood as triacylglycerols and phospholipids (PLs). Oral administration of PL DHA forms, *sn*-1 lysophosphatidylcholine-DHA (*sn*-1 LPC-DHA) and di-DHA phosphatidylcholine (di-DHA-PC), has been suggested to increase brain DHA levels by ∼100% (relative percent) and up to ∼200% (concentration) compared with controls. In contrast, triacylglycerol-DHA and nonesterified-DHA do not produce increases in brain DHA when provided in the diet. However, a subsequent study using a higher dose of *sn*-1 LPC-DHA did not confirm these findings and reported no significant increase in brain DHA. To address these inconsistencies, we aimed to replicate previous investigations of PL-DHA forms (LPC and PC) and their impact on brain DHA levels. Mice were randomly divided into one of four groups and received a daily gavage for 30 days of 80 μl of either corn oil alone (control) or corn oil containing 1 mg of DHA as nonesterified DHA, *sn*-1 LPC-DHA, or di-DHA-PC. DHA relative percent and concentrations were determined in brain regions (cortex, cerebellum, hippocampus, amygdala, striatum, and remainder of brain) and plasma using GC-flame ionization detection. Following treatment, no significant differences in DHA relative percent or concentration were observed between control and/or treatment groups in any brain region. Relative percent of plasma DHA was significantly elevated in all DHA-treated groups compared with the control group, confirming systemic absorption of the supplemented DHA. Our results demonstrate that dietary DHA provided as *sn*-1 LPC-DHA or di-DHA-PC does not increase brain DHA levels compared with nonesterified-DHA or the control group, failing to reproduce prior reports.

DHA (22:6n-3) is a long-chain omega-3 (n-3) PUFA that constitutes approximately 40% of total PUFA in the brain, where it plays critical roles in maintaining membrane fluidity, supporting synaptic signaling, and facilitating neuroprotection (1, 2). Because the brain has limited capacity to synthesize DHA from its precursor α-linolenic acid (ALA; 18:3n-3), it relies on the uptake of preformed DHA from peripheral tissues and plasma pools, derived from the diet or endogenous synthesis (1, 2, 3, 4, 5). In the diet, DHA is primarily found in fish and seafood, where it exists mainly in esterified forms such as triacylglycerols (TAGs) and phospholipids (PLs).

Upon ingestion, dietary DHA esterified to TAG is hydrolyzed by pancreatic lipase in the small intestine, producing NEFA and 2-monoacylglycerol. These digestion products are re-esterified into TAG, and packaged into chylomicrons (CMs), which circulate in the blood to deliver fatty acids to peripheral tissues. CM remnants, containing lipids like DHA, are transported to the liver where they are processed and repackaged into lipoproteins for secretion into the bloodstream. PL forms of DHA undergo a similar process, being hydrolyzed into NEFA and lyso-PL intermediates before being repackaged into CM. Dietary DHA in the form of lysophosphatidylcholine (LPC) would be re-esterified to phosphatidylcholine (PC) within enterocytes and incorporated into the lymphatic circulation as part of CM (6). Once delivered to peripheral tissues, DHA can be released into the plasma pool either as NEFA or LPC associated with albumin or liver-secreted lipoproteins, allowing transport to the brain to maintain brain DHA levels (7).

Among the various dietary forms of DHA, PL-bound species, including *sn*-1 lysophosphatidylcholine-DHA (1-docosahexaenoyl-2-hydroxy-*sn*-glycero-3-phosphocholine [*sn*-1 LPC-DHA also referred to as sn-1 DHA-LPC]), and di-DHA phosphatidylcholine (1,2-didocosahexaenoyl-sn-glycero-3-phosphocholine [di-DHA-PC]), have been reported to substantially increase brain DHA levels more effectively than TAG-DHA or nonesterified-DHA (NE-DHA) when consumed in the diet, as demonstrated in a series of influential studies by Subbaiah *et al.* (8, 9, 10, 11). In one study, oral administration of *sn-1* LPC-DHA (1 mg DHA/day) to adult mice for 30 days resulted in a 2-fold (∼100%) increase in brain DHA relative percent, and an estimated 2- to 3-fold (100–200%) increase in DHA concentration, depending on the brain region, whereas the same dose of NE-DHA had no effect on brain DHA levels (8). In another study, rats were dosed orally with *sn*-1 LPC-DHA for 30 days, which led to a 100% increase in brain DHA, whereas TAG-DHA failed to elevate brain DHA levels (9). Furthermore, di-DHA-PC, which yields LPC-DHA during digestion, also increased brain DHA by ∼35% (9). Similarly, lipase-treated krill oil, which generates LPC-DHA from the dietary PL-DHA form, was reported to increase brain DHA by 5-fold in wild-type mice compared with untreated krill oil (10). In a follow-up study using APOE3- and APOE4-targeted replacement mice, lipase-treated krill oil elevated DHA levels in both plasma and hippocampus. Hippocampal DHA levels were approximately 1.5- to 1.7-fold (50–70%) higher than controls depending on sex and APOE genotype (11).

The proposed mechanism by Subbaiah *et al.* suggests that when DHA occupies the *sn*-1 position of the LPC, it can be absorbed intact and transported directly to the brain, bypassing the conventional CM route and delivery to peripheral tissues (8, 10). However, we are not aware of any direct evidence supporting the intact absorption of *sn*-1 DHA into the plasma (6). Furthermore, a recent study by Andriambelo *et al.* tested the efficacy of *sn*-1 LPC n-3 supplementation in APOE3 and APOE4 knock-in mice, using doses substantially higher than those in the study by Sugasini *et al.* (9.6 mg/day vs. 1.0 mg/day) and for a longer duration (4 months vs. 30 days) and found no significant increases in brain DHA levels, even when compared with a low n-3 PUFA control group, regardless of genotype or duration (12). Given the magnitude of the previously reported effects and their potential therapeutic implications, it is essential to confirm whether PL forms of DHA (LPC and PC) lead to a drastic increase in brain DHA levels. Thus, we aimed to directly replicate previous work conducted by Sugasini *et al.* (8, 9) to evaluate whether the substantial increase in brain DHA following either *sn*-1 LPC-DHA or di-DHA-PC could be reproduced with C57BL/6J mice under rigorously matched dietary and environmental conditions.

## Materials and Methods

### Materials

NE-DHA, *sn*-1 LPC-DHA, and di-DHA-PC were purchased from Larodan AB (Solna, Sweden). The fatty acid internal standard, docosatrienoic acid (22:3n-3) ethyl ester, and the GC reference standard (GLC-462) were purchased from NuChek Prep, Inc (Elysian, MN). Boron trifluoride in methanol (14%) was purchased from Sigma-Aldrich (St Louis, MO). All solvents used were American Chemical Society or HPLC grade and were purchased from either MilliporeSigma (Mississauga, ON, Canada) or Fisher Scientific (Ottawa, ON, Canada).

### Animals

**Fig. 1:**
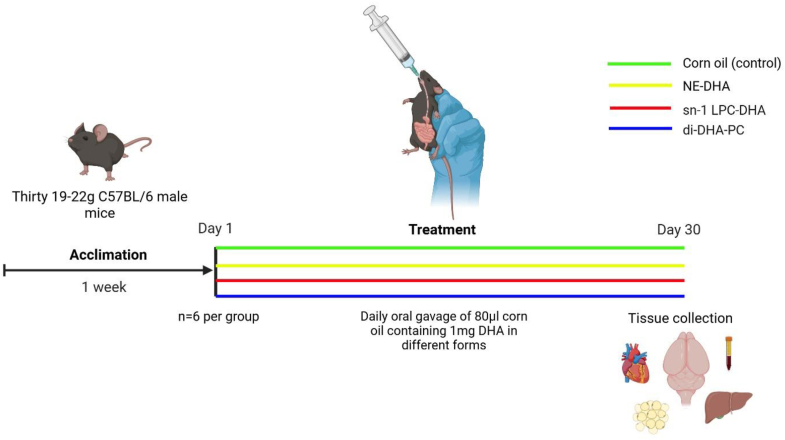
A total of 30 male C57BL/6J mice were used. Six mice were sacrificed prior to gavage treatments to obtain baseline measurements. The remaining 24 mice were randomly divided into four groups (n = 6 per group) and received a daily gavage of 80 μl of either corn oil alone (control) or treatment groups containing corn oil with 1 mg of DHA as NE-DHA, *sn*-1 LPC-DHA, or di-DHA-PC for 30 days. After an overnight fast, mice were humanely euthanized, and brain regions (cortex, cerebellum, hippocampus, amygdala, striatum, and ROB), liver, heart, adipose tissue, and blood were collected.

The University of Toronto Animal Ethics Committee approved the experimental animal protocol (protocol #20013069), which was conducted in accordance with the policy and guidelines of the Canadian Council on Animal Care and the Regulations of Animals Research Act of Ontario. Throughout the study, food and water were available ad libitum, and substantial care was taken to minimize animal suffering. Male C57BL/6J mice (Jackson Laboratories) weighing 19–22 g were housed in rooms with a 12 h light-dark cycle and controlled temperature (22 ± 2°C). The mice were acclimated for 1 week before starting the daily gavage and were provided rodent chow (Teklad LM 485; Envigo, Indianapolis, IN) throughout the experiment, in addition to the daily gavage of corn oil containing the various DHA preparations. A total of 30 male C57BL/6J mice were used. Six mice were sacrificed prior to gavage treatments to obtain baseline measurements. The remaining 24 mice were randomly divided into four groups (n = 6 per group) and received a daily gavage of 80 μl of either corn oil alone (control) or treatment groups containing corn oil with 1 mg of DHA as NE-DHA, *sn*-1 LPC-DHA, or di-DHA-PC for 30 days (Fig. 1). After an overnight fast, animals were anesthetized with isoflurane, and blood was collected from the left ventricle prior to euthanasia by intracardiac perfusion. To minimize residual blood in tissues, a 5-min perfusion with cold saline was performed. Brain (cortex, cerebellum, hippocampus, amygdala, striatum, and remainder of brain [ROB]), liver, heart, adipose, and blood were collected, flash frozen in liquid nitrogen, and stored at −80°C until biochemical processing.

### Gavage oil preparations

Oil preparations for gavage were adapted from methods described by Sugasini *et al.* (9). Each DHA compound (NE-DHA, *sn*-1 LPC-DHA, and di-DHA-PC) and control (80 μl corn oil) were first dissolved in ethanol and then added dropwise to corn oil while stirring for 15 min at room temperature. The ethanol was then evaporated under a stream of nitrogen, and the resulting oil mixtures were stored at 4°C to prevent oxidation. Prior to administration (1.0 mg/day), oils were brought to room temperature and thoroughly mixed. Fresh dispersions were prepared every 4 days to ensure consistency and stability.

### Lipid extraction and methylation

Total lipids were extracted from brain tissues and plasma by modified methodologies adapted from Folch *et al.* (13). Cortex, cerebellum, hippocampus, amygdala, striatum, and ROB (∼25, ∼50, ∼20, ∼3, ∼8, and ∼280 mg, respectively) were homogenized with 6 ml 2:1 chloroform:methanol (v:v) and 1.75 ml of 0.88% aqueous potassium chloride buffer to separate lipid-containing and aqueous phases. A known mass of docosatrienoic acid ethyl ester (22:3n-3; NuChek Prep, Inc) was added for fatty acid quantification, and samples were vortexed thoroughly and left exposed to extraction solvents at 4°C for 24 h. For the cortex, one sample from the di-DHA-PC group was excluded from analysis due to physical damage prior to fatty acid extraction. In the striatum, one NE-DHA sample was removed due to a poor-quality chromatogram with abnormally low peak intensities, which prevented reliable quantification. Additionally, two samples were excluded from the hippocampal analysis, one from the NE-DHA group and one from the di-DHA-PC group, due to insufficient tissue collected during dissection, reducing the sample size to n = 5 for each of these groups. Following the 24-h extraction period, the lipid-containing chloroform phase was isolated. An aliquot of the extract was dried under a stream of nitrogen for most brain regions; however, for the amygdala and striatum, 100% of the lipid extract was dried down and used for transesterification. Lipids were extracted from plasma using methods described previously for brain tissue; however, plasma (∼40 μl) was added to 3 ml 2:1 chloroform:methanol (v:v), and samples were immediately vortexed without a 24-h extraction, after which 1.75 ml of 0.88% aqueous potassium chloride was added. For the plasma, 100% of the dissolved lipids were dried down under nitrogen. Transesterification to fatty acid methyl esters (FAMEs) was performed by the addition of 1 ml of 14% boron trifluoride in methanol (MilliporeSigma, Burlington, MA) and 0.3 ml hexane and heating in an oven for 1 h at 100°C. After samples were cooled to room temperature, 1 ml of milliQ H_2_O and 1 ml of hexane was added. The samples were vortexed, centrifuged at 500 *g*, and the upper hexane layer was separated. This layer was dried under nitrogen, reconstituted in 100 μl of heptane, and stored in GC vials for analysis.

### FAME quantification

FAMEs were quantified via GC-flame ionization detection (GC-FID) using a SCION 8300 GC (SCION Instruments, Goes, NL) equipped with a DB-FFAP 15 m × 0.10 mm i.d. × 0.10 μm film thickness, nitroterephthalic acid modified, polyethylene glycol, capillary column (J&W Scientific from Agilent Technologies, Mississauga, ON). One microliter of FAME mixture was introduced into the injector with the SCION 8400 Pro autosampler. The injector was heated to 250°C, with split ratios of 30:1 for ROB, 60:1 for cortex, hippocampus, and amygdala, and 100:1 for cerebellum and striatum. The column oven temperature was programmed initially at 150°C for 0.25 min followed by a temperature increase of 35°C/min to 200°C and then 4°C/min to 225°C with a 2.07 min hold time. Finally, the temperature was increased at a rate of 80°C/min to 245°C and maintained at this temperature for 13 min. The FID detector was set at 300°C. Air and nitrogen make up gas flow rates were 300 and 30 ml/min, respectively. FID sampling frequency was set at 100 Hz. The identity of chromatogram peaks was determined by comparing the peak retention times to an external mixed FAME standard (GLC-462; Nu Chek Prep) using Compass CDS software. Concentrations of individual FAMEs were quantified by comparing the area under the curve of each FAME peak to that of the internal standard (22:3n-3), and were corrected for the internal standard mass and the tissue weight or volume.

### Statistics and calculations

All reported data are expressed as means ± SE. The sample size ranged from five to six mice per time point throughout the study. A one-way ANOVA with Tukey’s multiple comparisons test was used to compare differences in DHA concentrations and relative percent between the groups. A *P* value less than 0.05 was considered statistically significant. All statistical analyses were conducted using GraphPad Prism, version 9.2.0 (GraphPad Software, Inc).

## Results

### Tissue fatty acid levels of C57BL/6J mice after 30-day gavage

**Fig. 2:**
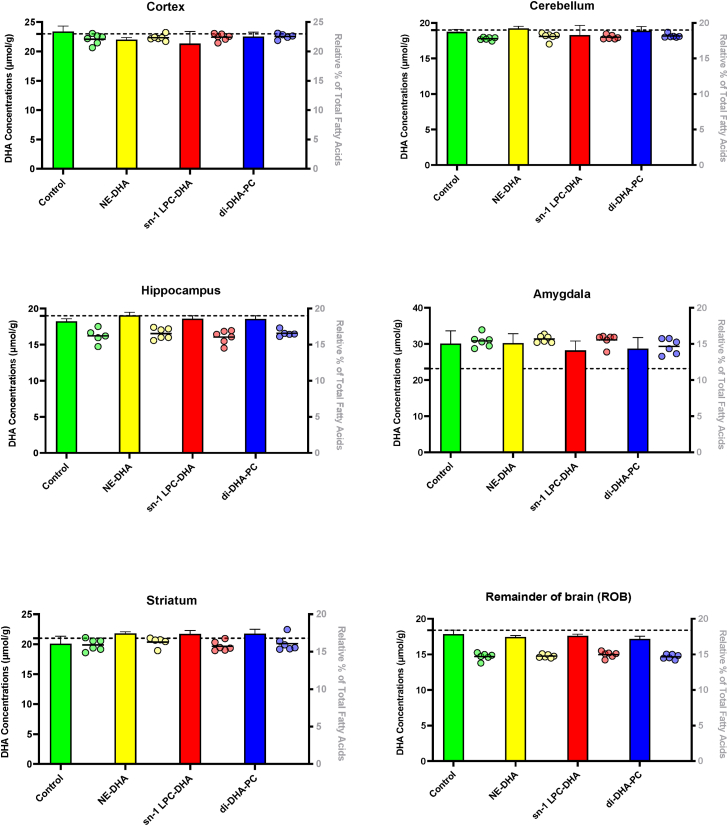
DHA relative percent (dots) and concentration (bars) in various brain regions of C57BL/6J mice following a daily gavage for 30 days with 80 μl of either corn oil alone (control) or corn oil containing 1 mg of DHA in the form of NE-DHA, *sn*-1 LPC-DHA, or di-DHA-PC (n = 5–6 per group). No significant differences in DHA relative percent or concentration were observed across treatment groups in any brain region (*P* > 0.05; one-way ANOVA). Baseline DHA concentrations are represented by the dashed line with the black band.

**Fig. 3:**
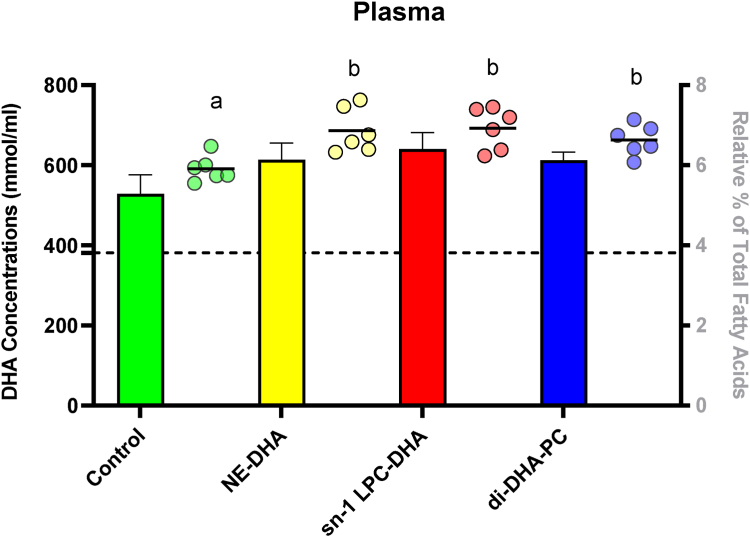
DHA relative percent (dots) and concentration (bars) in plasma of C57BL/6J mice following a daily gavage for 30 days with 80 μl of either corn oil alone (control) or corn oil containing 1 mg of DHA in the form of NE-DHA, *sn*-1 LPC-DHA, or di-DHA-PC (n = 6 per group). Dots in each panel that do not share a common letter differ significantly (*P* < 0.05; one-way ANOVA, post hoc Tukey’s test). Baseline DHA concentrations are represented by the dashed line with the black band.

Brain regions (cortex, cerebellum, hippocampus, amygdala, striatum, and ROB), plasma, liver, heart, and adipose were collected from male C57BL/6J mice at baseline and upon receiving a daily gavage of 80 μl of either corn oil alone (control) or corn oil containing 1 mg of DHA as NE-DHA, *sn*-1 LPC-DHA, or di-DHA-PC (n = 5–6 per group) for 30 days (Fig. 1). DHA concentrations and relative percent of brain regions are presented in Figure 2, and plasma DHA concentrations and relative percent are presented in Figure 3. Liver, heart, and adipose DHA concentrations are displayed in supplemental Fig. S1.

Following 30 days of daily gavage (80 μl), no significant differences in DHA relative percent or concentration were observed between control and treatment groups in any brain region (*P* > 0.05; one-way ANOVA). Mean baseline DHA concentrations were as follows: cortex, 23 μmol/g; cerebellum and hippocampus, 19 μmol/g; amygdala, 23 μmol/g; striatum, 21 μmol/g; and ROB, 18 μmol/g. Furthermore, the mean baseline plasma DHA concentration was 380 nmol/ml but did not differ between DHA treated groups. The relative percent of plasma DHA was significantly elevated in all DHA-treated groups compared with the control group (*P* = 0.0029; one-way ANOVA, post hoc Tukey’s test), whereas plasma DHA concentration did not differ significantly between groups (*P* = 0.2317; one-way ANOVA). No significant differences in DHA concentration were observed in the liver (*P* = 0.0913). The concentration of DHA in the heart was significantly elevated in all DHA-treated groups compared with the control group (*P* = 0.0157; one-way ANOVA, post hoc Tukey’s test) but did not differ between DHA treatments. Adipose DHA concentration differed significantly among groups (*P* = 0.0191), with higher levels in the NE-DHA group versus control (*P* = 0.0166; one-way ANOVA, post hoc Tukey’s test), but no differences were detected between DHA treatments. See supplemental Fig. S1 for liver, heart, and adipose concentrations.

## Discussion

The current study set out to resolve conflicting findings in the literature regarding the ability of LPC and PC forms of DHA to increase brain DHA levels. Specifically, we aimed to replicate the findings of Sugasini *et al.* (8), who reported a 2-fold (100%) increase in brain DHA relative percent following dietary gavage with *sn*-1 LPC-DHA, along with a 2- to 3-fold (∼100–200%) increase in DHA concentration, varying by brain region. To test these results, we replicated their experimental design, including mouse weight, strain and supplier (19–22 g male C57BL/6J mice [Jackson Laboratories]), background rodent chow (Teklad LM 485), treatment groups, DHA dose and preparation (1 mg DHA/day), gavage vehicle (corn oil), duration of treatment (30 days), housing conditions (lighting/temperature), overnight fasting prior to tissue collection, as well as tissue perfusion and tissue collection procedures. Despite matching these variables, we observed no significant differences in DHA concentration or relative percent in any brain region between control and treatment groups. Plasma DHA relative percent was elevated in all DHA-treated groups, confirming systemic absorption, despite the low dose of 1 mg DHA/day (∼0.2% of total dietary fat). However, this increase did not translate into higher brain DHA levels, regardless of the form of DHA administered. Importantly, DHA concentration in the heart was significantly increased in all DHA-treated groups, indicating tissue uptake consistent with the plasma response. Our findings align with those of Andriambelo *et al.*, who also failed to detect increases in brain DHA following *sn*-1 LPC-DHA supplementation in both APOE3 and APOE4 replacement mice after 4 months, despite using a much higher dose (9.6 mg/day), longer duration (2–4 months), and an n-3 PUFA-deprived control (12). Notably, when DHA was expressed as a percentage of total fatty acids, Andriambelo *et al.* did observe a 22% increase in APOE3 mice after 2 months of LPC n-3 supplementation, though this effect was not sustained at 4 months. As with our study, it remains unclear why both studies failed to reproduce the rather large increase in brain DHA reported by Sugasini *et al.* (8).

While we attempted to control as many variables as possible, our study is not without limitations. While we observed a significant increase in the relative percent of plasma DHA in all DHA-treated groups, the plasma DHA concentration did not differ significantly (*P* = 0.2317). This apparent discrepancy might be explained by the relatively low dose of DHA used in the study, because concentrations tend to be more variable than relative percent, and we might be underpowered for plasma concentrations, as we powered the study for brain DHA levels. Nevertheless, increases in the plasma relative percent of DHA in all treatment groups compared with the control support the bioavailability of all treatments.

Although we only measured total DHA content in the brain to replicate the study by Sugasini *et al.*, prior findings show that radiolabeled NE-DHA and LPC-DHA are differentially incorporated into specific brain PL subclasses upon intravenous administration (14). Future work examining lipid class-specific DHA incorporation would help improve our understanding of how dietary DHA sources influence brain lipid levels. Our sample size (n = 5–6 per group) was slightly lower than that of the study by Sugasini *et al.* (n = 6–8 per group); however, a priori power calculations indicated that our study was powered to detect differences in brain DHA of the magnitude previously reported (8). Therefore, this modest difference is unlikely to account for the dramatic increase in brain DHA levels. It is difficult to conceive of experimental variables that might differ between studies in a way that would explain the magnitude of brain DHA enrichment observed by Sugasini *et al.*, especially given that the major variables known to affect DHA concentrations other than dietary DHA (i.e., sex, background linoleic acid, and ALA in the diet) (15, 16, 17) were matched in our study and even then only led to relatively small differences in DHA levels. However, there are several discrepancies in the studies by Subbaiah *et al.* worth mentioning. In their first report, as we have discussed previously (18, 19), concentrations of brain DHA in mice fed corn oil (with background chow containing ALA) were about 40-fold lower than commonly reported values in the literature with similar dietary fatty acid intake (∼0.5 vs. ∼20 μmol/g). Furthermore, DHA concentrations per brain mass in phosphatidylethanolamine were much higher than total lipid DHA, which is not plausible. Subsequent studies of Subbaiah *et al.* no longer report concentrations and instead only report relative percentages; thus, it is more difficult to assess whether the results are plausible. In another study, LPC-eicosapentaenoic acid was administered to mice at 3.3 μmol/day for 15 days, which resulted in a doubling of brain DHA (20). Assuming the mouse brain contains 20 μmol of DHA per gram of tissue, this would suggest that about 5–10% of the dose of EPA was not only converted to DHA but also accreted in the brain, an amount that is orders of magnitude higher than previous reports of brain DHA accretion/uptake (21, 22, 23), let alone conversion from eicosapentaenoic acid to DHA and accretion upon dietary intake. Lastly, while Subbaiah *et al.* hypothesize that *sn*-1 DHA is the form that increases brain DHA more than *sn*-2 DHA, in Fig. 5 of their earlier article (8), *sn*-2 DHA increases brain DHA more than *sn*-1 DHA in two brain regions (amygdala and striatum), with the remaining three regions being not statistically different. Given the ongoing debate over the stability and digestibility of *sn*-1 DHA (24, 25), compounded by conflicting data on which form preferentially enriches brain DHA, we also attempted to replicate their study using di-DHA-PC in mice. However, again we failed to reproduce the finding by Subbaiah *et al.* of increased brain DHA upon administration of di-DHA-PC.

In summary, our results do not support the claim that PL-DHA forms significantly increase brain DHA levels under rigorously matched experimental conditions and are consistent with those reported by Andriambelo *et al.* (12).

## Data availability

All datasets generated during and/or analyzed during the current study are available from the corresponding author on reasonable request.

## Supplemental data

This article contains supplemental data.

## Conflict of interest

R. P. B. has received industrial grants, including those matched by the Canadian government, and/or travel support from Arctic Nutrition, Bunge Ltd, DSM, The Dairy Farmers of Canada, Mead Johnson, Natures Crops International, Nestec, Inc, Pharmavite, and Sansero Life Sciences, Inc. R. P. B. has served as a consultant to Bunge Ltd, Fonterra, and Red Abbey Labs. Moreover, R. P. B. was on the executive committee of the International Society for the Study of Fatty Acids and Lipids and held a meeting on behalf of fatty acids and cell signaling, both of which rely on corporate sponsorship. R. P. B. has given expert testimony in relation to supplements and the brain. A. H. M. is on the Board of Directors of the International Society for the Study of Fatty Acids and Lipids, is a Science Advisor for Benexia and Natures Crops International, and is a coapplicant on a joint government/industry-funded research grant with Natures Crops International. All other authors declare that they have no conflicts of interest with the contents of this article.
