# Supplementary material for: Dietary phospholipid carriers of DHA do not increase brain DHA levels: a replication study
Source: J Lipid Res. 2025 Sep 26;66(11):100913. doi: 10.1016/j.jlr.2025.100913 (PMC12684751; doi:10.1016/j.jlr.2025.100913)
Supplement: Supplementary fig 1 [file mmc1.docx]

**SUPPLEMENTAL INFORMATION:**

**Dietary phospholipid carriers of DHA do not increase brain DHA levels: A replication study**

Brinley J. Klievik^1^, Yan Fu^1^, Aidan D. Tyrrell^1^, Chuck T. Chen^1^, Adam H. Metherel^1^, and Richard P. Bazinet,^1^

^1^Department of Nutritional Sciences, Temerty Faculty of Medicine, University of Toronto, 1 King’s College Circle, Toronto, Ontario, Canada, M5S 1A8

**Supplemental Fig. S1:** DHA concentration in liver, heart, and adipose tissue of C57BL/6J mice following a daily gavage for 30 days with 80 µL of either corn oil alone (control) or corn oil containing 1 mg of DHA in the form of NE-DHA, sn-1 LPC-DHA, or di-DHA-PC (n = 5-6 per group). Bars in each panel that do not share a common letter differ significantly (p < 0.05; one-way ANOVA, Tukey's post hoc test). Baseline DHA concentrations are represented by the dashed line with the black band. *DHA, docosahexaenoic acid; NE-DHA, non-esterified DHA; sn-1 LPC-DHA, 1-docosahexaenoyl-2-hydroxy-sn-glycero-3-phosphocholine; di-DHA-PC, 1,2-didocosahexaenoyl-sn-glycero-3-phosphocholine*
